# Considerations When Designing Inclusive Digital Health Solutions for Older Adults Living With Frailty or Impairments

**DOI:** 10.2196/63832

**Published:** 2024-10-21

**Authors:** Emilie Kauffeldt Wegener, Jenny M Bergschöld, Sverre Bergh, Ad van Berlo, Camilla Wong Schmidt, Afroditi Konidari, Lars Kayser

**Affiliations:** 1 Department of Public Health University of Copenhagen København K Denmark; 2 SINTEF Trondheim Norway; 3 Innlandet Hospital Trust Ottestad Norway; 4 Smart Homes Eindhoven Netherlands; 5 Region Zealand Holbæk Denmark; 6 Tendertec Cardiff United Kingdom; 7 Department of Public Health University of Copenhagen Copenhagen Denmark

**Keywords:** digital health services, frameworks, sociotechnical ecosystem, older adults, co-design

## Abstract

This viewpoint is written by authors with industrial, clinical, and academic backgrounds within medical and social sciences. The purpose is to share our experiences with digital health innovation from a sociotechnical perspective. The audience for the viewpoint is innovators, researchers, service designers, and project managers with little or some experience with theory-informed programs, complex interventions, and implementation or reorganization of sociotechnical ecosystems in health care. In digital health innovation projects, barriers related to traditions and cultures among researchers, clinicians, and industry may arise. Moreover, the final digital solutions may not always fit into existing digital ecosystems and may thus require a reorganization of how health care is provided at horizontal and vertical levels. The collaborating researchers have experience working in the field of digital health innovation for more than a decade, and we have developed and used 4 frameworks and models that are particularly relevant for theory-based complex interventions and can be used to inform inclusive co-design of digital health solutions with a sociotechnical perspective. These are (1) the 4E, a matrix to include, engage, empower, and emancipate marginalized people; (2) the GO-TO model, which can be used as a design navigator; (3) the Epital Care Model, to inform infrastructure; and (4) the Readiness and Enablement Index for Health Technology instrument, to stratify service users. From January 2021 to September 2024, we had the opportunity to apply these into practice in 4 living labs located in Denmark, Norway, the Netherlands, and Canada as a part of a European Union–funded project on “Smart Inclusive Living Environments.” The goal was to cocreate a digital solution and reorganize health care services to reduce social isolation, increase health literacy, and enhance well-being for older adults living with frailty or impairments. Based on our experiences with the Smart Inclusive Living Environments project, we have formed a proposal for how design guidelines for sociotechnical innovation projects can be structured, backed up with reflections based on our experiences. With that, design guidelines should include three areas: (1) a common vocabulary including theories, frameworks, and models; (2) templates and protocols for methods, including detailed guidelines and templates for the planned development of the technologies; and (3) methods to implement and provide education and training of service users and informal and formal caregivers. In the design process, we emphasize the importance of involving relevant stakeholders in the implementation of the created design guidelines to obtain preparedness in the organizations, as well as including putative service users to ensure the likelihood of adoption. Moreover, it is important to align expectations, have a common understanding of the applied frameworks and methods, and have access to the necessary resources to reach successful results.

## Background

The increased use of digital health services in health care, defined by the National Institute of Health as “the use of information and communications technologies in medicine and other health professions to manage illnesses and health risks and to promote wellness” [1], is often seen as the way to support the 3-fold global challenge of an increased number of older adults with long-term health conditions, an increased number of older adults in need of specialized care, and an ongoing reduction in the health care workforce [2,3]. It is anticipated that digital health services can help empower patients by enabling self-management and providing increased access to knowledge, thus enabling them to make more informed decisions in managing their health both in and outside traditional health care settings, and offering a more holistic view of patient’s health condition for health care professionals due to increased access to a plentitude of data [4]. To be successful in achieving these ambitions, it is important that new digital health services are developed to be safe, ethical, secure, reliable, equitable, and sustainable in their design [3]. Thus, the development of new digital services should ideally follow the principles of transparency, accessibility, scalability, replicability, privacy, security, interoperability, and confidentiality [3]. To fulfill this at national, regional, and local levels, it is essential to involve multiple stakeholders, that is, the industry that develops and markets the services; the buyers; and the end users, such as patients or citizens and formal and informal caregivers. This calls for systematic innovative approaches including frameworks, guidelines, and principles for design processes and models for the assessment of digital services [5-7].

Today, there is an increased focus on involving end users, as well as on how to and to what extent this involvement should be carried out in a design process, to address their needs, values, and perspectives to ensure adaptability and sustainable solutions [4,7,8]. But despite this recognition, there are still significant knowledge gaps that need to be addressed. In a recent review, we found three knowledge gaps in the current scientific literature: (1) older adults with cognitive impairments are often excluded from design processes; (2) recruitment through partners is the most used method, which holds the risk of recruitment bias; and (3) participants are not included in all phases of a design process [8].

Another important aspect within the field of co-designing new digital health services is the risk of excluding marginalized groups, particularly those who are disconnected, disengaged, or disempowered, such as older adults living alone with a limited social network [9]. Another aspect to address when discussing co-designing digital services is the implementation process. In this process, it is important to ensure transparency, safety, security, and sustainability and that the involved stakeholders’ needs and preferences are addressed before implementing the solution. Moreover, the emphasis should be on training and onboarding of all stakeholders, especially the health care professionals, that is, registered nurses, physicians, and other allied health care professionals using and promoting the use of the technology for the target groups [10].

For partners in academia and small- and middle-sized enterprises, the many frameworks, methods, and legal requirements may seem overwhelming and may, therefore, constitute a barrier to the collaborative development of new digital solutions. To help overcome potential barriers and ensure the development of accessible, meaningful, and sustainable digital services, this viewpoint is based on experiences from a 45-month European Union–Canadian project, Smart Inclusive Living Environments (SMILE) [11]. SMILE was a multistakeholder project that aimed to co-design a sociotechnical ecosystem and digital solution with older adults living with frailty or impairments, increase overall well-being, and reduce social isolation. Based on our experiences, we present what we find important to include as content for design guidelines for sociotechnical ecosystems in digital health design processes.

A major challenge in the design of new technologies for older adults with frailty or impairments is the sociotechnical clash between the world of technology and the everyday life of older adults [12]. Focusing on the everyday life of older adults, and not merely on their diagnosis, enables a broad understanding of their needs and preferences from a holistic perspective [13]. This would perhaps not be possible if the technology was meant for a group of patients living with a specific diagnosis, since the privilege of the design would be given to the medical perspective. A holistic approach increases the likelihood of developing a solution that is scalable and transferable and that can be used across different diseases and patient groups [7]. Therefore, the SMILE project focused on how to include and identify the needs of older adults with frailty or impairments; ensure inclusion in all phases of the design process, from the initial problem identification to the final implementation and evaluation; and increase the likelihood of adoption and sustainable use of the technology among the users.

The project undertook a holistic approach and built on earlier frameworks used to organize the provision of cross-sectoral health care, identify segments of users, and inform design processes. In the following, we provide suggestions based on our experiences, backed up with Multimedia Appendices 1-5, providing a more detailed insight into the theories and frameworks we used, an example of a vocabulary, recruitment considerations, design methods, and consideration in relation to implementation and setting up training and education.

## Our Experiences With Person-Centered Design for a Sociotechnical Ecosystem

When initiating a complex project, it is important to consider whether the project should be based on theory-informed programs and then agree upon the theories and frameworks to be used. Another relevant aspect in the initial phase of the design process is to consider the Medical Research Council (MRC) guidelines for complex interventions [14]. The SMILE design process was informed by the ECM framework with adaptations from a regional Danish project, PreCare [15]; the Readiness and Enablement Index for Health Technology (READHY) instrument informed by Optimise Health Literacy and Access [16]; and the intervention map by Bartholomew et al [17].

Moreover, the partners in SMILE decided to use theories and frameworks to organize the work. These were based on work by one of the authors (LK) and national and international researchers. These theories and frameworks are the 4E framework for coproducing digital services to engage, empower, and emancipate disengaged people living with complex and chronic conditions [9]; the GO-TO model, from goal to outcome [18]; the Epital Care Model (ECM) [19]; and the READHY instrument [20]. We also illustrate how principles from these frameworks have been further developed in the project, for example, how text vignettes can be developed based on the READHY instrument (A Kelly, 2024, unpublished data). These frameworks and models are further described in Multimedia Appendix 1. Further, the SMILE project was informed by literature identified prior to and during the SMILE project, as well as findings from a scoping review on how to involve older adults with frailty or impairments in the design of digital health technologies to enable aging in place, which was conducted as part of the project [8].

## Context

The SMILE project [11] was a partnership across 4 living labs in Canada, Denmark, the Netherlands, and Norway involving health care organizations, research institutions, and small- and middle-sized enterprises. The work in SMILE aimed to enable older adults to live an independent and active life, irrespective of frailty or physical and cognitive impairments. To reach this aim, we designed a digital platform with and for older adults and their informal caregivers. We also established a digital service catalog, for vendors to help integrate their products into the SMILE ecosystem.

## A Common Vocabulary

At the beginning of the project, it was clear that we did not have a common language within the research field, due to our multidisciplinary backgrounds. Therefore, we developed a vocabulary to be used in our communication within the project. The vocabulary was meant to facilitate our communication and avoid misunderstandings enabling us to work toward a common goal and purpose. We believe that a common language is important in a development and design process where several stakeholders are involved, including in the development of personal technologies and digital services; that is, industrial partners need to be guided based on the same concepts, frameworks, and standards. The common vocabulary was formed based on the most used and profound terms within the project. These included frailty, impairment, service users, formal and informal caregivers, participants, investigators, and living labs. Our vocabulary is presented in Multimedia Appendix 2.

Overall, we found that a common vocabulary supported meaningful conversations and enhanced the process of writing research articles in multidisciplinary teams. It was not possible to fully align the language throughout the project despite this effort; however, we would still recommend that projects composed of multidisciplinary teams, cross-sectoral settings, and various geographic locations to develop a mutual understanding of concepts and terms to avoid lengthy discussions.

## Our Considerations About Inclusion, Recruitment, Ethics, and Data Management

When initiating a complex project, it is important to consider who the target group is and how to ensure their integrity as individuals, for example, in cases where the target group is individuals living with moderate to severe cognitive impairments. It is also important to consider how eligibility criteria are defined, consider how to ensure representation, and include individuals that are not normally given a voice in development processes. Finally, ethical aspects and considerations regarding data management should also be considered and well defined, especially when working in an international project where different laws and regulations have to be addressed. Our considerations on these parameters are further elaborated in Multimedia Appendix 3.

## Design Process

An important aspect when initiating a co-design process is to start by defining relevant methods to select and stratify participants for the co-design process, as well as to initiate the process of developing initial user requirement specifications. An outline of the methods considered and applied in the SMILE project is presented in Multimedia Appendix 4.

## Implementation

When the digital health solution has been developed and is ready for testing, the implementation phase is initiated. Several aspects should be considered in the implementation phase, especially when designing a digital solution to be integrated into a sociotechnical ecosystem with horizontal and vertical integration, where existing roles and responsibilities will be affected. This may also involve changed workflows or revision of regional, national, and international guidelines. In the implementation phase, we found it important to focus on the interoperability of the designed digital services to ensure compatibility when expanding the sociotechnical ecosystem, to avoid creating or consolidating existing silo systems. In Multimedia Appendix 5, considerations and solutions related to the SMILE project are reported.

Prior to and during the implementation, we found that both formal and informal caregivers need training to gain skills to promote the use of new services and technologies, as they often play an important role in relation to the successful adoption of technologies among service users. There is increasing attention on how to educate and train formal and informal caregivers [2,21]. Many of these programs including the basic certification for ECM staff, focused on technology and communication skills [15]. This is important to ensure confidence among caregivers; avoid stress and anxiety; make them be aware of their role; and help them better understand the service users’ needs, preferences, and values [22,23].

It is important that education and training are adapted to the tasks and competence of the involved caregivers and organization. All vendors are required to provide documentation and material to train relevant stakeholders in the technology provided. During the development of the dashboard, 1 lesson was that embedding the training in the design of the solution supports sustained users and retained skills. As users become more proficient in navigating and using technologies, they can enhance their ability to provide care.

## Considerations Based on Our Experiences

In this viewpoint, we have presented how theory-informed frameworks can be used as a scaffold for a multistakeholder project. A novelty of our shared experiences in this viewpoint is that this is the first publication in an international scientific journal that operationalizes ECM and READHY to guide the development of an inclusive, digitally enabled environment with a focus on the active involvement of service users and formal and informal caregivers. Moreover, only a few projects designing digital solutions for older adults living with frailty or impairments have been successful in including participants in all phases of the design process, from the initial problem identification to the final implementation and evaluation [8]. The presentation of how we have successfully handled these challenges, including a reflection on specific challenges, is a novel contribution to the abundant literature on how to develop and evaluate digital health services.

## Why This Viewpoint?

Large-scale projects often need leadership and transparent planning. Many organizations and industrial partners have their own principles for how to conduct projects. Often, *Projects IN Controlled Environments, 2nd Edition* or tailored versions are used [18]. Although *Projects IN Controlled Environments, 2nd Edition* is an effective scalable model, it may be difficult to use in complex settings such as European Union projects based on cross-country collaboration, aimed toward several goals and on achieving key performance indicators. The challenge in such a setting is often to align mindset, cultural understanding, and experience from different organizations of health care provision into a joint effort. These challenges may also limit the use of a specific digital health design framework [6] to ensure common standards and user experiences in designing an ecosystem.

In this context, we found that several conversations among all partners helped us to align our understanding of concepts, terminologies, frameworks, and theories enabled by our common vocabulary. These conversations initiated ongoing dialogue, for example, about using the term client versus patient or citizen, or how to describe the involved actors’ roles, responsibilities, technologies, and location of each formal or informal caregiver.

## Academic Perspective

Investigators in SMILE came from different disciplines, ranging from the positivistic evidence-based medical field with a background in medicine or nursing and engineering, to social science and humanities based on more phenomenological approaches. This combination of experienced investigators fostered relevant discussions regarding various disciplines’ methodologies. We recommend projects to have these important discussions inspired by our considerations. In this context, it should be noted that we have not worked with behavioral change theories such as the Health Belief model [24], the self-determination theory [25], or the Precaution Adoption Process Model [26]; instead, we were inspired by the 4E matrix’s 3D thinking, involving the micro, meso, and macro levels, and the presentation by Bødker et al [27] on vertical and horizontal integration to understand how to include a group of informants with different skills, to understand their context and the new context for the developed technologies. This was an important prerequisite for our understanding of the implementation and how to educate and train the involved actors. In this process, it was a great help to the Norwegian living lab that the Danish living lab had documented the results of their 2-year, action-based research and implementation of the ECM [15]. Other projects may have other purposes, such as to change the health behavior of a specific population, and therefore need to be inspired by other frameworks. Thus, each project needs to address what theories suit their purpose.

## A Complex Intervention

The context of the project is living labs, constituting a sociotechnical health-focused ecosystem. The project is a complex intervention, inspired by program theory and recognizes the importance of digital services as a part of service design. The project could have followed the MRC’s guidelines for complex interventions more strictly [28]. However, with the project being based on a collaboration between academic researchers, engineers, clinical researchers, clinicians, and industrial partners toward the common goal of developing new digital services and implementing a new structure for digital health, the project is grounded in a context than the one that the MRC framework suggests. There are, however, similarities, as SMILE builds on theory development and evaluation in the design. The presented design guidelines may thus be useful for projects that use program theory in their intervention and build on the MRC guidelines for complex interventions. In this way, the design guidelines contribute to the existing literature on design principles, such as technical documents and usability guidelines, by bridging the theory-informed academic approach with an empirically grounded sociotechnical dimension.

## Limitations

The context of the technology design presented in this viewpoint was development up to technology readiness level 6-7 [29], and the focus was, therefore, limited to documenting the development of individual technologies to ensure certification and meet requirements, for example, from the UK National Institute of Healthcare Excellence’s evidence standard framework for digital health and care technologies [30]. The use of the design guidelines in other projects or technology readiness levels remains to be established, but we do not have any indications from our work that this could be a problem. It should be noted that the context primarily has been older adults living with chronic obstructive pulmonary disease or with cognitive impairments. The design guidelines should, therefore, be expanded upon in settings involving other disease and age groups. Moreover, the experiences of using the methods are limited to investigators in 4 countries—Canada, Denmark, the Netherlands, and Norway—and participants were required to understand the official national languages. This is due to the high costs associated with using interpretations and translation of validated evaluation instruments in the design phases. It should, however, always be considered how documents, design activities, and evaluation instruments can be translated to avoid cultural exclusion. All living labs in the SMILE project are in higher-income countries. Thus, no lower- or middle-income countries are represented. This calls for a validation of the design guidelines in projects in low- and middle-income countries. In this context, it is advantageous that the models—GO-TO, ECM, and READHY—are agile and suitable for projects with fewer resources or access to digital infrastructure.

## Final Remarks

This study presents views on important considerations to do when initiating a digital design process involving older adults with frailty or impairments. The views are based on experiences from a cross-disciplinary project involving academic researchers, clinicians, industrial partners, and engineers. The study stresses the importance of forming a common vocabulary; provides reflections on how to involve different user groups; and suggests various frameworks, for example, the GO-TO model, ECM, and REAHY, that have been proven to work successfully when involving older adults with frailty or impairments and moderate to severe cognitive impairments in an innovative project. These experiences may help inform future projects by building upon, expanding, or adapting them to a specific context, for example, by adding or replacing theories and vocabulary.

## Appendix

Multimedia Appendix 1 Models and frameworks used in the Smart Inclusive Living Environments (SMILE) project.

Multimedia Appendix 2 Example of a vocabulary.

Multimedia Appendix 3 Inclusion, recruitment, ethics, and data management.

Multimedia Appendix 4 Considerations about the design process.

Multimedia Appendix 5 Considerations about implementation.

## Abbreviations

ECM: Epital Care Model
MRC: Medical Research Council
READHY: Readiness and Enablement Index for Health Technology
SMILE: Smart Inclusive Living Environments
