# Supplementary material for: Considerations When Designing Inclusive Digital Health Solutions for Older Adults Living With Frailty or Impairments
Source: JMIR Form Res. 2024 Oct 21;8:e63832. doi: 10.2196/63832 (PMC11535789; doi:10.2196/63832)
Supplement: Multimedia Appendix 1 [file formative_v8i1e63832_app1.docx]

### The Framework for the Co-production of Digital Services That Engage, Empower and Emancipate Marginalised People Living with Complex and Chronic Conditions (4E)

The 4E matrix (1) is inspired by a socio-technical model by Botin et al (2) with focus on digital health as a mean to avoid exclusion and engage, empower, and emancipate individuals who are disconnected, disengaged and disempowered and in risk of being marginalized. The matrix suggests how to stepwise mitigate barriers related to use of digital healthcare services and thereby ensuring that more people get access and can gain the advantages from the digitalization. The matrix addresses both horizontal and vertical processes and emphasize the necessity of work processes at an individual, interpersonal, and organizational level. It calls for activities to identify and engage individuals that are not known to the healthcare sector and find ways to include them. When the individuals are included, the aim is to create a trajectory for each of them, in collaboration with relevant caregivers, supported at a structural level. The model thus proposes theories and concepts of engagement and empowerment e.g., addressing health literacy at the individual level as a prerequisite for being empowered (3).

### From Goal to Outcome to inform design (GO-TO model)

The GO-TO model was developed in response to the need of a navigator to inform innovation of tele-health services at a community or hospital level. The GO-TO model is an intuitive tool that builds on project models and process-oriented health technology assessment models and does not require certification to apply [11]. The GO-TO model consists of four phases, 1) inception, 2) materialization, 3) implementation, and 4) evaluation. For each phase several steps are defined to help navigate the phases. For Inception focus is to identify stakeholders, identify available relevant technologies define a clear goal for the project, form a budget and ensure that resources are available. In the materialization phase, focus is on whether new technologies should be developed, existing be modified or off the shelf solutions can be acquired and configured to the project. In the implementation phase focus is on education and training of users, and on process evaluation to ensure progression in the project. This phase also includes communication and dissemination results as well as activities to inform a business model. Finally, the planned outcome is evaluated using an assessment model e.g., the Model for assessment of Telemedicine (MAST) [19,20] or equivalent. In SMILE an adaptation of the MAST for municipalities was used and modified [21]. The model is not intended for development of complex digital health solutions nor suitable for informing the expansion of socio-technical systems. As many components will be familiar to people who have used the MAST to evaluate telehealth-based projects, or used health technology assessment tools or process evaluation, like several partners in SMILE, it was possible to use the GO TO model in the four SMILE living labs with only a short introduction and with virtual support.

### The Epital Care Model for Service Design

The ECM is the conceptual framework used to establish the living labs in SMILE. The ECM consists of six levels and can be used as a template for how digital health services can be utilized efficiently, following the service users’ varying health condition. The ECM support organizing activities and resources by mapping each actors’ roles, responsibilities, context, and how a digital service is utilized in each ECM level. This can support efficient workflows and timely care.

The ECM design principles can facilitate an understanding of needs and requirements for people living with one or more LTHC (4,5). The principles of ECM is to inform cross-disciplinary and cross-organizational provision of healthcare, to be agile, efficiently utilize resources, and provide timely care, near the service users, based on patient reported data such as activity tracking, and other monitored data.

The model is designed to be proactive, preventive, and monitor the healthcare system that involves individuals in managing their health conditions. All levels from ECM1 to ECM6 are connected and transitions between levels are gated by specific events or triggers. These events are communicated to a response and coordination center (RCC) based on data reported by the service user or data acquired and reported directly through wearables or other digital tools used by the service user to the RCC. For each service user a filter is defined based on disease specific variables defined by medical specialists. With that, thresholds for each variable are introduced based on health measures when the service user is in the habitual state. Since the habitual state may decline over time, the personalized threshold is adjusted at routine checks with healthcare professionals every 3, 6 or 12 months. The change in condition may lead to jumps between the levels. In this way, the transitions are not necessarily sequential (4,6,7).

In SMILE, focus was on ECM1 and 2, due to a focus on service users ‘digitally facilitated active and independent living’ and how they can be assisted in managing their condition, i.e., by using different health technologies, be engaged, and supported by the RCC (5), to mitigate referral to acute healthcare services. In ECM1, focus is on how service users can be educated and trained to manage their health condition, learn about condition-specific challenges, and engage in activities that may improve their physical, mental, and social well-being. The education and training activities aim to increase health literacy, empowerment, and self-management (8). An approach in designing activities and involving service users is to meet them in a coaching and facilitating way where the technology facilitates learning, empowerment, and self-management, and does not constitute a prerequisite in the level of understanding, skills, and literacy. SMILE’s design process was inspired by Maathuis et al.’s value service design (9), as well as the 4E (1) to ensure that all involved actors, particularly healthcare professionals and the health organizations, focus on addressing the older adults’ values and ensure representation in the involvement e.g., by not only including older adults who are active, well-educated, empowered, engaged and outgoing. This can be assured by recruiting participants through a variety of channels including non-digital media and community-based activities (10).

### The readiness and enablement index for health technology (READHY)

The READHY framework is build based on the e-health literacy framework (11) and the eHealth literacy questionnaire (eHLQ) (12) supplemented with four dimensions from the health education impact questionnaire (heiQ) (13) to assess self-management, and two dimensions from the health literacy questionnaire (HLQ) (14). Thus, READHY consists of 13 dimensions from heiQ, HLQ, and eHLQ that can be used in combination to describe a user’s health technology readiness level and degree of enablement (12). The instrument can be used both qualitatively (15–17) through text vignettes describing patient groups based on their READHY scores, and quantitatively addressing the values of the scores. Finally, data from READHY combined with other data e.g., about health condition and socio-demographic characteristics such as age, gender, educational level, or digital behavior can be used to create profiles within groups of people. These profiles can be used to ensure recruitment of participants with different characteristics to take part in design and research activities (17,18). This can help ensure inclusion of representatives from various segments and not only the usual suspects.

Using READHY and how to stratify data is described in section three.

## Living labs

The concept of a living labs goes back to the 18^th^ century and the work of Pasteur (19). Today a living lab is often described as a user-centred, open ecosystem operating in a territorial context e.g., a community or a region (20), based on a systematic user co-creation approach in public–private–people partnerships, integrating research and innovation processes in real life communities and settings (21,22). A particular strength of a living lab is that it is isolated from existing structures, economic models, and policies. This isolation of living labs enables challenging existing societal structures by providing arenas suitable for testing new and disruptive ideas (19). In SMILE, we draw on this opportunity to explore and exploit how our living lab environments in Canada, Denmark, the Netherlands, and Norway can facilitate new ways of interaction, and improved health and well-being among older adults with frailty or impairments.

**References**

1. Kayser L, Nøhr C, Bertelsen P, Botin L, Villumsen S, Showell C, m.fl. Theory and practice in digital behaviour change: A matrix framework for the co-production of digital services that engage, empower and emancipate marginalised people living with complex and chronic conditions. Informatics [Internet]. 2018;5(4). Tilgængelig hos: https://www.scopus.com/inward/record.uri?eid=2-s2.0-85060920898&doi=10.3390%2finformatics5040041&partnerID=40&md5=ccf0028da04923e9fd1e4c9f9b0dace1

2. Botin L, Bertelsen PS, Kayser L, Turner P, Villumsen S, Nøhr C. People Centeredness, Chronic Conditions and Diversity Sensitive eHealth: Exploring Emancipation of the ‘Health Care System’ and the ‘Patient’ in Health Informatics. Life. 7. december 2020;10(12):329.

3. Kayser L, Karnoe A, Duminski E, Somekh D, Vera-Muñoz C. A new understanding of health related empowerment in the context of an active and healthy ageing. BMC Health Serv Res. december 2019;19(1):242.

4. Phanareth K, Vingtoft S, Christensen AS, Nielsen JS, Svenstrup J, Berntsen GKR, m.fl. The Epital Care Model: A New Person-Centered Model of Technology-Enabled Integrated Care for People With Long Term Conditions. JMIR Res Protoc. 16. januar 2017;6(1):e6.

5. Schmidt CW, Borgnakke K, Frølich A, Kayser L. Preferences, Needs, and Values of Patients With Chronic Obstructive Pulmonary Disease Attending a Telehealth Service: Qualitative Interview Study. JMIR Hum Factors. 21. juni 2024;11:e53131.

6. Blueprint. PRECARE: The PreCare clinic - A sector-neutral, data-driven, and person-centred healthcare service [Internet]. 2024. Tilgængelig hos: https://smileehealth.eu/knowledge-and-learning-from-smile-precare/

7. Phanareth K, Dam AL, Hansen MABC, Lindskrog S, Vingtoft S, Kayser L. Revealing the Nature of Chronic Obstructive Pulmonary Disease Using Self-tracking and Analysis of Contact Patterns: Longitudinal Study. J Med Internet Res. 19. oktober 2021;23(10):e22567.

8. Lindskrog S, Christensen KB, Osborne RH, Vingtoft S, Phanareth K, Kayser L. Relationship Between Patient-Reported Outcome Measures and the Severity of Chronic Obstructive Pulmonary Disease in the Context of an Innovative Digitally Supported 24-Hour Service: Longitudinal Study. J Med Internet Res. 2. juni 2019;21(6):e10924.

9. Maathuis I, Niezen M, Buitenweg D, Bongers IL, van Nieuwenhuizen C. Exploring Human Values in the Design of a Web-Based QoL-Instrument for People with Mental Health Problems: A Value Sensitive Design Approach. Sci Eng Ethics. april 2020;26(2):871–98.

10. Oberschmidt K, Grünloh C, Tunç S, van Velsen L, Nijboer F. You can’t always get what you want: Streamlining stakeholder interests when designing technology-supported services for Active and Assisted Living. I: 32nd Australian Conference on Human-Computer Interaction [Internet]. Sydney NSW Australia: ACM; 2020 [henvist 7. februar 2022]. s. 649–60. Tilgængelig hos: https://dl.acm.org/doi/10.1145/3441000.3441040

11. Norgaard O, Furstrand D, Klokker L, Karnoe A, Batterham R, Kayser L, m.fl. The e-health literacy framework: A conceptual framework for characterizing e-health users and their interaction with e-health systems. Knowl Manag E-Learn Int J. 15. december 2015;522–40.

12. Kayser L, Rossen S, Karnoe A, Elsworth G, Vibe-Petersen J, Christensen JF, m.fl. Development of the Multidimensional Readiness and Enablement Index for Health Technology (READHY) Tool to Measure Individuals’ Health Technology Readiness: Initial Testing in a Cancer Rehabilitation Setting. J Med Internet Res. 12. februar 2019;21(2):e10377.

13. Osborne RH, Elsworth GR, Whitfield K. The Health Education Impact Questionnaire (heiQ): An outcomes and evaluation measure for patient education and self-management interventions for people with chronic conditions. Patient Educ Couns. maj 2007;66(2):192–201.

14. Osborne RH, Batterham RW, Elsworth GR, Hawkins M, Buchbinder R. The grounded psychometric development and initial validation of the Health Literacy Questionnaire (HLQ). BMC Public Health. december 2013;13(1):658.

15. Kikkenborg J, Magelund E, Riise MS, Kayser L, Terp R. Knowledge, Skills, and Experience With Technology in Relation to Nutritional Intake and Physical Activity Among Older Adults at Risk of Falls: Semistructured Interview Study. JMIR Hum Factors. 8. maj 2024;11:e52575.

16. Terp R, Kayser L, Lindhardt T. Older Patients’ Competence, Preferences, and Attitudes Toward Digital Technology Use: Explorative Study. JMIR Hum Factors. 14. maj 2021;8(2):e27005.

17. Nielsen AS, Appel CW, Larsen BF, Kayser L, Hanna L. Patient perspectives on digital patient reported outcomes in routine care of inflammatory bowel disease. J Patient-Rep Outcomes. december 2021;5(1):92.

18. Thorsen IK, Rossen S, Glümer C, Midtgaard J, Ried-Larsen M, Kayser L. Health Technology Readiness Profiles Among Danish Individuals With Type 2 Diabetes: Cross-Sectional Study. J Med Internet Res. 15. september 2020;22(9):e21195.

19. Hesseldal L, Kayser L. Healthcare innovation – The Epital: A living lab in the intersection between the informal and formal structures. Qualitative Sociology Review. 2016;12(In print).

20. Chesbrough HW. Open innovation: the new imperative for creating and profiting from technology. Boston, Mass: Harvard Business School Press; 2003. 227 s.

21. Bilgram V, Brem A, Voigt KI. USER-CENTRIC INNOVATIONS IN NEW PRODUCT DEVELOPMENT — SYSTEMATIC IDENTIFICATION OF LEAD USERS HARNESSING INTERACTIVE AND COLLABORATIVE ONLINE-TOOLS. Int J Innov Manag. september 2008;12(03):419–58.

22. Pallot M. Engaging Users into Research and Innovation: The Living Lab Approach as a User Centred Open Innovation Ecosystem. 2009;
