# Supplementary material for: Considerations When Designing Inclusive Digital Health Solutions for Older Adults Living With Frailty or Impairments
Source: JMIR Form Res. 2024 Oct 21;8:e63832. doi: 10.2196/63832 (PMC11535789; doi:10.2196/63832)
Supplement: Multimedia Appendix 2 [file formative_v8i1e63832_app2.docx]

Travers et al [1] and Glegg et al [2], describe frailty as “a state of physiological vulnerability with diminished capacity to manage external stressors.” The prevalence amongst people aged 65+ is approx. 11% and 80+ years approx. 50% [1]. To better understand the frailty level of older people, frailty measures can be used, such as “The Clinical Frailty Scale” [3,4].

Impairment refers to older adults with physical or mental conditions that cause disability, that can be addressed or compensated for through social or technological means [5].

The term service users are widely used in the United Kingdom both by the National Health Services (NHS) and in telemedicine studies such as the whole system demonstrator [6,7]. In SMILE service users refers to older adults who are currently enrolled in and provided with relevant healthcare services in the SMILE living labs.

Caregiver is a term commonly used for those providing any kind of health and care services. Formal caregivers are used for healthcare professionals and informal caregivers are used to address family, friends, and individuals affiliated with NGOs [8].

The term participants are used for those who have given consent to participate in any part of the SMILE field studies and design process.

Investigators, refers to those who collect data through the administration of questionnaires, interviews, observations, workshops, or other methods. Investigators should be invited to take part in handling and reporting the collected data and offered co-authorship in publications.

Living labs are often described as a user-centred, open ecosystem operating in a territorial context e.g., a community or a region and are often isolated from existing structures, economic models, and policies.

**References**

1. Travers J, Romero-Ortuno R, Bailey J, Cooney MT. Delaying and reversing frailty: a systematic review of primary care interventions. Br J Gen Pract. januar 2019;69(678):e61–9.

2. Glegg SMN, Holsti L, Stanton S, Hanna S, Velikonja D, Ansley B, m.fl. Evaluating change in virtual reality adoption for brain injury rehabilitation following knowledge translation. Disabil Rehabil Assist Technol. 2017;12(3):217–26.

3. Fournaise A, Nissen SK, Lauridsen JT, Ryg J, Nickel CH, Gudex C, m.fl. Translation of the updated clinical frailty scale 2.0 into Danish and implications for cross-sectoral reliability. BMC Geriatr. december 2021;21(1):269.

4. Church S, Rogers E, Rockwood K, Theou O. A scoping review of the Clinical Frailty Scale. BMC Geriatr. december 2020;20(1):393.

5. Lindsay S, Brittain K, Jackson D, Ladha C, Ladha K, Olivier P. Empathy, participatory design and people with dementia. I: Proceedings of the 2012 ACM annual conference on Human Factors in Computing Systems - CHI ’12 [Internet]. Austin, Texas, USA: ACM Press; 2012 [henvist 24. juni 2021]. s. 521. Tilgængelig hos: http://dl.acm.org/citation.cfm?doid=2207676.2207749

6. Hirani SP, Rixon L, Beynon M, Cartwright M, Cleanthous S, Selva A, m.fl. Quantifying beliefs regarding telehealth: Development of the Whole Systems Demonstrator Service User Technology Acceptability Questionnaire. J Telemed Telecare. maj 2017;23(4):460–9.

7. Omeni E, Barnes M, MacDonald D, Crawford M, Rose D. Service user involvement: impact and participation: a survey of service user and staff perspectives. BMC Health Serv Res. december 2014;14(1):491.

8. Kayser L, Karnoe A, Duminski E, Somekh D, Vera-Muñoz C. A new understanding of health related empowerment in the context of an active and healthy ageing. BMC Health Serv Res. december 2019;19(1):242.
