# Supplementary material for: Considerations When Designing Inclusive Digital Health Solutions for Older Adults Living With Frailty or Impairments
Source: JMIR Form Res. 2024 Oct 21;8:e63832. doi: 10.2196/63832 (PMC11535789; doi:10.2196/63832)
Supplement: Multimedia Appendix 3 [file formative_v8i1e63832_app3.docx]

### **An inclusive way to recruit and Involve Service Users**

Recruitment can be carried out through different methods. A series of methods were identified in a scoping review conducted in SMILE. These include, purposeful sampling methods, outreach approaches, and inviting participants through partners (1). The main source of recruitment in SMILE was ‘invitation through partners’, i.e., through the living labs PreCare, Innlandet Hospital Trust and TanteLouise. In recruiting participants, several factors are relevant to consider. Service users in living labs, such as in SMILE, can be gated by healthcare professionals’ assumptions and tendency to protect older adults with frailty or impairments. Thereby, sampling bias can occur, which results in a skewed impression of the challenges, needs, and desires of the broad population of older adults. Disadvantaged, disempowered, and disconnected older adults may be overlooked, including people from lower- and middle-income (LMIC) settings, even though they are likely to benefit most from these services (2–4).

Another example of recruitment bias is recruiting individuals who are active and known to the people recruiting them i.e., participants with a high level of digital health literacy, patient representatives or individuals who are active within patient associations. This group is already part of a community, connected, engaged, and empowered. It is important to avoid these types of recruitment bias, it should therefore be discussed how the living labs can recruit to include disconnected, disengaged, and disempowered people as proposed by Kayser, et al. 2018 (2) and Lindsay et al. (37). In the following a list of methods to recruit participants not known to the healthcare professionals is provided: 1) Recruitment through public call for participants, e.g., through digital or printed flyers (7), 2) recruitment in public places, or senior gathering places, such as parks, squares, and malls (8), 3) recruitment through participation in local support groups or patient organizations for older adults with frailty or impairments (9,10). To ensure a broad range of perspectives it is recommended to recruit via many channels and ensure inclusion from communities that are not commonly accessible by the formal caregivers. The READHY instrument together with other socio-demographic determinants can also be used to ensure recruitment of service users with different levels of capacity to self-manage, levels of social support, and digital health literacy. This will ensure that participants in the design and evaluation of services and technologies are broadly represented. In the process, the navigator from goal to outcome (GO-TO), may also help plan the involvement, ensure that resources are available and that the planned design does not already exists (11).

The process of recruiting for the SMILE field studies started after the responsible care institutions, and the investigators agreed on how-, and how many participants should be recruited. The means to recruit however differed within SMILE, according to the maturity level of the living lab. PreCare in Denmark and the Living lab in Norway was mature, and here recruitment was done in collaboration with the registered nurses on site. TanteLouise in the Netherlands had a different focus. They are in a transition towards digital care by keeping older adults with moderate cognitive impairment at home, longer than usual; so, recruitment was done through internal sessions or community events among every new patient, that would normally enter one of their nursing homes.

For future projects we recommend that representatives from each living lab fill out a document describing possible sampling bias and ethical considerations about how the service users are recruited. Participants recruited through consent from another person holding a power of attorney should be included as an entity. In each living lab, participatory observations of the healthcare professionals’ practice of recruiting participants should be conducted, to explore how the recruitment unfolds, and possibly positively or negatively affects the representativeness of the participants. In living labs where the healthcare professionals are both investigators, and responsible for recruiting, investigators from other living labs should conduct the participatory observations.

### **Eligibility Criteria**

In conducting and preparing for multicenter studies, it is important to work based on common eligibility criteria. In SMILE these criteria cover, excluding people with an expected lifetime of less than three months, people diagnosed with psychiatric conditions with psychotic episodes, and people who lack the skill to understand the language that the interactions and services offer.

Participants should not be excluded due to impairments that regularly occur in an older population, e.g., mild, or moderate cognitive impairments, visual or hearing impairments, or other physical impairments such as paresis. Participants should be able to give informed consent themselves, with help from a formal caregiver, or an informal caregiver that holds a power of attorney, according to local ethical approvals. In the latter case, the participant should be offered to express their consent orally or in writing, despite the lack of legal validity.

### **Ethics and Data Management Considerations**

All research activities should be managed according to outlined ethical principles within the project, and to local ethical regulations and approvals. In SMILE all data are handled according to the General Data Protection Regulation (GDPR) (12) in Denmark, Norway, and the Netherlands, and according to guidelines by the Hamilton Integrated Research Ethics board (HiREB) in Canada (13). Data handling was conducted according to the EU Open Science policy (14). Each investigator is to ensure that; all relevant ethical permits are obtained from appropriate instances, that informed consent is obtained and that there is a local plan for data management and storage that aligns with the projects data management plan. In SMILE each country had different rules for protocols, and this delayed some of the processes such as sharing data or planning site studies by partners located in another country. We thus designed the studies to have data collected and analyzed locally to avoid sharing person or health-related data across institutions.

**References**

1. Wegener EK, Bergschöld JM, Whitmore C, Winters M, Kayser L. Involving Older People With Frailty or Impairment in the Design Process of Digital Health Technologies to Enable Aging in Place: Scoping Review. JMIR Hum Factors. 27. januar 2023;10:e37785.

2. Kayser L, Nøhr C, Bertelsen P, Botin L, Villumsen S, Showell C, m.fl. Theory and practice in digital behaviour change: A matrix framework for the co-production of digital services that engage, empower and emancipate marginalised people living with complex and chronic conditions. Informatics [Internet]. 2018;5(4). Tilgængelig hos: https://www.scopus.com/inward/record.uri?eid=2-s2.0-85060920898&doi=10.3390%2finformatics5040041&partnerID=40&md5=ccf0028da04923e9fd1e4c9f9b0dace1

3. Hart JT. The inverse care law. Lancet Lond Engl. 27. februar 1971;1(7696):405–12.

4. Showell C, Turner P. The PLU problem: are we designing personal ehealth for people like us? Stud Health Technol Inform. 2013;183:276–80.

5. Lindsay S, Brittain K, Jackson D, Ladha C, Ladha K, Olivier P. Empathy, participatory design and people with dementia. I: Proceedings of the 2012 ACM annual conference on Human Factors in Computing Systems - CHI ’12 [Internet]. Austin, Texas, USA: ACM Press; 2012 [henvist 24. juni 2021]. s. 521. Tilgængelig hos: http://dl.acm.org/citation.cfm?doid=2207676.2207749

6. Kayser L, Nøhr C, Bertelsen P, Botin L, Villumsen S, Showell C, m.fl. Theory and Practice in Digital Behaviour Change: A Matrix Framework for the Co-Production of Digital Services That Engage, Empower and Emancipate Marginalised People Living with Complex and Chronic Conditions. Informatics. 9. november 2018;5(4):41.

7. Hassan L, Swarbrick C, Sanders C, Parker A, Machin M, Tully MP, m.fl. Tea, talk and technology: patient and public involvement to improve connected health ‘wearables’ research in dementia. Res Involv Engagem. december 2017;3(1):12.

8. Jacelon C, LeBlanc R, Alkhawaldeh M, Ridgway J, Marquard J, Choi J. Developing ASSISTwell, a tablet application to support older adult’s self-management of symptoms of chronic conditions. Gerontechnology. 16. april 2018;17(1):18–28.

9. Hakobyan L, Lumsden J, O’Sullivan D. Participatory Design: How to Engage Older Adults in Participatory Design Activities. Int J Mob Hum Comput Interact. juli 2015;7(3):78–92.

10. de Barros AC, Cevada J, Bayés À, Alcaine S, Mestre B. User-centred design of a mobile self-management solution for Parkinson’s disease. I: Proceedings of the 12th International Conference on Mobile and Ubiquitous Multimedia - MUM ’13 [Internet]. Lule&#229;, Sweden: ACM Press; 2013 [henvist 7. februar 2022]. s. 1–10. Tilgængelig hos: http://dl.acm.org/citation.cfm?doid=2541831.2541839

11. Kayser L, Phanareth K. Leave no one behind: the role of digital health literacy. I: Pinnock H, Poberezhets V, Drummond D, redaktører. Digital Respiratory Healthcare [Internet]. Sheffield, United Kingdom: European Respiratory Society; 2023 [henvist 2. februar 2024]. s. 79–94. Tilgængelig hos: http://erspublications.com/lookup/doi/10.1183/2312508X.10001023

12. THE EUROPEAN PARLIAMENT AND OF THE COUNCIL. REGULATION (EU) 2016/679 OF THE EUROPEAN PARLIAMENT AND OF THE COUNCIL of 27 April 2016 on the protection of natural persons with regard to the processing of personal data and on the free movement of such data, and repealing Directive 95/46/EC (General Data Protection Regulation) [Internet]. Official Journal of the European Union; 2016 [henvist 26. juni 2024]. Tilgængelig hos: https://eur-lex.europa.eu/legal-content/EN/TXT/PDF/?uri=CELEX:32016R0679

13. HiREB. Hamilton Integrated Research Ethics board [Internet]. 2022. Tilgængelig hos: https://hireb.ca/

14. European Comission. Open Science [Internet]. European Comission; 2022. Tilgængelig hos: https://research-and-innovation.ec.europa.eu/strategy/strategy-2020-2024/our-digital-future/open-science_en
