# Supplementary material for: Considerations When Designing Inclusive Digital Health Solutions for Older Adults Living With Frailty or Impairments
Source: JMIR Form Res. 2024 Oct 21;8:e63832. doi: 10.2196/63832 (PMC11535789; doi:10.2196/63832)
Supplement: Multimedia Appendix 4 [file formative_v8i1e63832_app4.docx]

### **The User Requirement Specification**

We find it important to develop a user requirement specification (URS) to align all project partners. An URS aims to address and identify service users’ needs and preferences in the initial phases of designing new digital technology. The URS was used to inform the intends, which were presented for vendors, developing the prototype of a conversational agent and help them to understand the contexts it was supposed to work within.

### **Stratification of Service users**

After participants are recruited, a stratification process can be initiated, to identify variations in the pool of participants based on their technology readiness, the challenges they face in everyday life and their needs and preferences, based on their network, self-management capabilities, and digital Health literacy (1–4). This is obtained by administering a questionnaire consisting of the READHY instrument (5) and socio-demographic questions including details of diagnosis and current use of technology. Registration of data and statistical analysis should take place at each local site. Anonymized data from all sites should be shared with one site for calculations aimed for comparison. The questionnaire should be distributed to as many service users as possible in each living lab.

### **Data Analysis Using READHY**

The process of stratifying participants based on data from the READHY questionnaires can be done in SPSS, R or STATA, by using the K-mean clustering algorithm. The number of clusters is usually between four and six, the choice of the final number of clusters is based on evaluation of; that the profiles or personas (6) within the clusters are meaningful from a clinical perspective, the socio-demographic characteristics constituting the population in each cluster, and the size of each cluster. In SMILE, stratification was made in four clusters based on data from the READHY questionnaire, in Norway and Denmark, using the K-mean clustering algorithm in R and SPSS (Bergh et al 2024, personal communication).

### **Iterative design process**

The design of the technology consists of several iteration processes including baseline interviews, workshops with partners, and three phases of co-creational workshops. The first process entailed the identification of needs, preferences, and current use of technologies among the service users. Initial baseline interviews were conducted at all sites. The baseline interviews were addressed in two different workshops among SMILE partners including a design workshop, and an ECM workshop in Denmark.

The iterative approach helped converge the initial ideas. In the development of the SMILEBot (7) we were able to narrow nine themes down to three functions, i.e., 1) my health, 2) daily activity, and 3) sleep. During the process it was obvious that the informal and formal caregivers also need digital assistance such as a dashboard. In Canada, Denmark, and Norway, this already existed in the software used for self-monitoring. In the Netherlands a dashboard was developed in SMILE in response to a need of connecting the technologies from various vendors, aiming to gather inputs from existing and coming digital tools in a way that made it easier for informal caregivers to people living with dementia (PWD) to access and thereby reduce their burden of care. The dashboard was developed in collaboration with informal caregivers to PWD and build on Tendertec’s existing commercial dashboard. The co-production activities in the Netherlands were structured within the context of “responsible innovation”. This approach focused on aligning the processes and outcomes of innovation with societal needs and concerns, requiring collaboration among diverse stakeholders including technology developers and users (8–11). This has meant not just addressing relevant health-related issues, but also ensuring that the technology fits within the dynamic context of unpaid caregivers and does not interfere with their everyday life and practices, but rather fits them. The user-centred approach involved considering the perspectives of both unpaid caregivers and project partners to identify the potential positive and negative impacts of the dashboard, especially focusing on ethically acceptable, sustainable, and socially desirable dimensions in the design process.

### **Identification of Challenges, Needs, and Desires**

In the first phase in SMILE the aim was to identify needs and desires based on knowledge about the participant's access to health information, network, available technologies, use of technology, and how their behaviour is influenced by their health and well-being.

To identify formal-, and informal caregivers and service users’ challenges, needs, and preferences, a combination of methods based on ethnography, anthropology, and service design is highly recommended, so data can be triangulated (12). Examples of such combinations can be found in Greenhalgh et al. (13) and Pradhan et al. (14). The overall idea of using different methods i.e., interviews, observations, and questionnaires, is that the users often do not know what they need. Some are just not aware of the underlying problems, other think they know a solution which is very specific to their own context, and some are not able to articulate their problems and needs.

### **Individual and focus group Interviews**

When processing data gathered through observations it's ideal to triangulate it with data gathered from individual or group interviews. Different materials can be used in these interviews, including photos taken by the participants, text vignettes, cueing cards, quotes, and sound clips to enhance ideation. Using activities and discussion stimuli might extend to including a demo of the designed technology. This enables users to provide detailed feedback. Ideally, interviews should be conducted in a setting that resembles a living room, with visual cues like a television, phone, tablet, or a Google assistant or Alexa hub in the room. Interviews should be conducted, transcribed, and analysed in the local language of each living lab site and then thematically summarized in English. All interviews and observations should be recorded and transcribed ad verbatim. All reports and transcriptions should be anonymized and shared with relevant stakeholders. Fieldwork material should also consist of notes, recordings, photos, and video if relevant and possible.

### **Participatory observations**

Participatory observation is a strong method to identify individuals challenges, needs, and preferences, e.g., in the home of the service user (15,16). Participatory observations may be helpful to understand how both healthcare professionals and service users respond to the implementation of new digital services and technologies. This knowledge can help inform the need for changes in workflows, and training and to identify potential stressful situations (17). Participatory observations may also help document unforeseen behaviours such as how healthcare professionals attitude as gatekeepers may influence their willingness to let specific service users participate in fieldwork activities, as they want to protect them (18). In SMILE participatory observations in the homes of the service users were successfully conducted with a focus on passive or active use of technology and digital services, as well as non-technical supported systems or interaction with others in relation to their health and well-being (12,17).

Data from participatory observations may also strengthen the validity for creating user journeys by providing the context of an everyday setting. Ethnographic methods and in particular auto-ethnography using video cameras (19) may help the investigator better understand everyday activities. If timelapse recordings are conducted with video cameras it is important to discuss the data on the recordings with the participants to avoid misinterpretation of the situations and activities. Finally, timelapse recordings or still photos may help focus and facilitate individual-, or group interviews. In SMILE the plan was to conduct auto-ethnographic studies, however, due to practical reasons, including post corona-pandemic lockdown and a fixed timeline, auto-ethnographic studies were not conducted.

### **Conducting workshops**

In workshop sessions we recommend having between four to eight participants present and if relevant, informal, or formal caregivers. The main objective of a workshop session is to encourage brainstorming and build trust and confidence among the participants, creating a safe space where they can share their experiences and thoughts related to healthcare services, formal and informal caregivers whether positive or negative. Workshop sessions can be used to generate ideas for future development or to verify the interpretation of data gathered from interviews and observations. Structuring the workshops with open questions can provide important input from participants. As such, they can function as an ideation room, an exploratory session, or a confirmatory session (14).

### **User Journeys**

User journeys refer to the sequence of events or specific tasks and interactions between stakeholders that an investigator observes over time and create maps of these observations (20) or instance, an investigator might observe how a person monitors their condition and interacts with others about it. User journeys can provide valuable insights into user behaviour and help investigators understand their needs and preferences. An alternative type of user journey may involve the implementation of new technologies as part of the living lab's services. This could also entail providing the service users with a prototype or dummy to observe how they interact with its features. Investigators may also employ videos featuring imaginary or invisible elements to induce concerns or advantages in an unfamiliar context or use Lego or modelling wax to stimulate creativity and ideation among participants (21–23).

### **User scenarios**

User scenarios is a method that can help describe e.g., the everyday life of service users before and after the introduction of new technology (24). This knowledge can help inform technology vendors in the mechanisms behind different needs and how the technology influences the service user’s behaviour. User scenarios in a design process should include service users with different levels of technology readiness. Here READHY can be used to help understand individuals’ skills, motivation, social interaction, burden of condition, and how this can impact the likelihood of adopting new technology. User scenarios can be made in collaboration with investigators and healthcare professionals. The scenarios can then be presented to vendors, who can use them to inform the development of profiles in the technology.

### **Involving people with cognitive impairment in the Design processes**

When involving people who suffer from psychotic episodes or with cognitive impairment there are factors to consider, and local ethics committees should be consulted. In SMILE we planned to involve people with moderate to severe cognitive impairment in the design process. In the following we present our considerations and suggestions related to this group and their informal caregivers based on experiences from the Netherlands and supplementary studies conducted in Denmark and Norway to identify how people with cognitive impairment can be involved. In SMILE we experienced that administration of questionnaires such as READHY to this population group can be difficult and requires support by trained persons. Conducting semi-structured interviews using the READHY framework to inform an interview guide may therefore be more meaningful in this setting (25). People with mild to moderate cognitive impairment can express their needs in individual interviews and should therefore be involved in problem and needs identification. Informal caregivers may take part and/or contribute but does not necessarily know what matters to them although they are aware of everyday challenges such as social interactions (Bergh et al., 2024 Personal communication).

In conducting interviews with people living with cognitive impairment using an empathetic approach to establish trust is beneficial. Involvement requires one-on-one interactions or involvement of informal caregivers, thus focus group interviews are not ideal (25).

A study within the SMILE project found that this group often needs solutions that are familiar to them, that involves the formal and informal caregiver’s and that focus on social interactions (25). In SMILE the contribution to the ecosystem for this group was a Dashboard to primarily be used by formal and informal caregivers connected to sensors in the home to be able to detect behavioural changes or irregularity of normal everyday patterns, including changes in circadian rhythm.

### **Data analysis**

Data collected through the named qualitative methods, may be analyzed as ethnographic material inspired by e.g., Spradley et al. (26). However, it is important to structure the empirical material in a way that helps the reader understand relevant parameters in terms of; the setting, context, situations in which a technology is present, typical patterns of activity, and who the participant interacts with or includes in their social sphere. These data may be triangulated and analysed using content analysis, thematic analysis, inductive, deductive, or abductive coding (27,28).

**References**

1. Kayser L, Rossen S, Karnoe A, Elsworth G, Vibe-Petersen J, Christensen JF, m.fl. Development of the Multidimensional Readiness and Enablement Index for Health Technology (READHY) Tool to Measure Individuals’ Health Technology Readiness: Initial Testing in a Cancer Rehabilitation Setting. J Med Internet Res. 12. februar 2019;21(2):e10377.

2. Rossen S, Kayser L, Vibe-Petersen J, Ried-Larsen M, Christensen JF. Technology in exercise-based cancer rehabilitation: a cross-sectional study of receptiveness and readiness for e-Health utilization in Danish cancer rehabilitation. Acta Oncol. 4. maj 2019;58(5):610–8.

3. Rossen S, Kayser L, Vibe-Petersen J, Christensen JF, Ried-Larsen M. Cancer Survivors’ Receptiveness to Digital Technology–Supported Physical Rehabilitation and the Implications for Design: Qualitative Study. J Med Internet Res. 5. august 2020;22(8):e15335.

4. Thorsen IK, Rossen S, Glümer C, Midtgaard J, Ried-Larsen M, Kayser L. Health Technology Readiness Profiles Among Danish Individuals With Type 2 Diabetes: Cross-Sectional Study. J Med Internet Res. 15. september 2020;22(9):e21195.

5. Kayser L, Rossen S, Karnoe A, Elsworth G, Vibe-Petersen J, Christensen JF, m.fl. Development of the Multidimensional Readiness and Enablement Index for Health Technology (READHY) Tool to Measure Individuals’ Health Technology Readiness: Initial Testing in a Cancer Rehabilitation Setting. J Med Internet Res. 12. februar 2019;21(2):e10377.

6. LeRouge C, Ma J, Sneha S, Tolle K. User profiles and personas in the design and development of consumer health technologies. Int J Med Inf. november 2013;82(11):e251–68.

7. Skjuve M, Følstad A, Brandtzæg PB. A Longitudinal Study of Self-Disclosure in Human–Chatbot Relationships. Interact Comput. 23. juni 2023;35(1):24–39.

8. Fraaije A, Flipse SM. Synthesizing an implementation framework for responsible research and innovation. J Responsible Innov. 2. januar 2020;7(1):113–37.

9. Owen R, Stilgoe J, Macnaghten P, Gorman M, Fisher E, Guston D. A Framework for Responsible Innovation. I: Owen R, Bessant J, Heintz M, redaktører. Responsible Innovation [Internet]. 1. udg. Wiley; 2013 [henvist 6. juni 2024]. s. 27–50. Tilgængelig hos: https://onlinelibrary.wiley.com/doi/10.1002/9781118551424.ch2

10. Von Schomberg R. A Vision of Responsible Research and Innovation. I: Owen R, Bessant J, Heintz M, redaktører. Responsible Innovation [Internet]. 1. udg. Wiley; 2013 [henvist 6. juni 2024]. s. 51–74. Tilgængelig hos: https://onlinelibrary.wiley.com/doi/10.1002/9781118551424.ch3

11. Schomberg R von, Hankins J, redaktører. International handbook on responsible innovation: a global resource. Cheltenham, UK Northampton, MA: Edward Elgar; 2019. 556 s.

12. Fetters MD, Curry LA, Creswell JW. Achieving Integration in Mixed Methods Designs-Principles and Practices. Health Serv Res. december 2013;48(6pt2):2134–56.

13. Greenhalgh et al. Making sense of technology adoption in healthcare: meso-level considerations. BMC Med. december 2015;13(1):92.

14. Pradhan A, Jelen B, Siek KA, Chan J, Lazar A. Understanding Older Adults’ Participation in Design Workshops. I: Proceedings of the 2020 CHI Conference on Human Factors in Computing Systems [Internet]. Honolulu HI USA: ACM; 2020 [henvist 7. februar 2022]. s. 1–15. Tilgængelig hos: https://dl.acm.org/doi/10.1145/3313831.3376299

15. Kayser L, Furstrand D, Nyman Rasmussen E, Monberg AC, Karnoe A. GoTO: A Process-Navigation Tool for Telehealth and -Care Solutions, Designed to Ensure an Efficient Trajectory from Goal Setting to Outcome Evaluation. Informatics. 12. september 2022;9(3):69.

16. Yock P, Zenios S, Makower J, Brinton TJ, Kumar UN, Watkins FTJ. Biodesign - The process of Innovating Medical Technologies. Second edition 2015. University Printing House, Cambridge CB2 8BS, United Kingdom: Cambridge University Press; 2015.

17. Schmidt CW, Borgnakke K, Frølich A, Kayser L. Preferences, Needs, and Values of Patients With Chronic Obstructive Pulmonary Disease Attending a Telehealth Service: Qualitative Interview Study. JMIR Hum Factors. 21. juni 2024;11:e53131.

18. Nielsen AS, Appel CW, Larsen BF, Hanna L, Kayser L. Digital patient-reported outcomes in inflammatory bowel disease routine clinical practice: the clinician perspective. J Patient-Rep Outcomes. december 2022;6(1):52.

19. Nourse R, Cartledge S, Tegegne T, Gurrin C, Maddison R. Now you see it! Using wearable cameras to gain insights into the lived experience of cardiovascular conditions. Eur J Cardiovasc Nurs. 14. oktober 2022;21(7):750–5.

20. Joseph AL, Kushniruk AW, Borycki EM. Patient journey mapping: Current practices, challenges and future opportunities in healthcare [Internet]. Knowledge Management & E-Learning: An International Journal (KM&EL); Tilgængelig hos: https://www.kmel-journal.org/ojs/index.php/online-publication/article/view/453

21. Wang T, Zhu H, Qian S, Giunti G, Goossens R, Melles M. Designing digital patient experiences: The digital health design framework. Appl Ergon. september 2024;119:104289.

22. Suijkerbuijk S, Nap HH, Cornelisse L, IJsselsteijn WA, de Kort YAW, Minkman MMN. Active Involvement of People with Dementia: A Systematic Review of Studies Developing Supportive Technologies. Baglio F, redaktør. J Alzheimers Dis. 18. juni 2019;69(4):1041–65.

23. Bødker S, Dindler C, Iversen OS. Tying Knots: Participatory Infrastructuring at Work. Comput Support Coop Work CSCW. april 2017;26(1–2):245–73.

24. Clemensen J, Larsen SB, Kyng M, Kirkevold M. Participatory Design in Health Sciences: Using Cooperative Experimental Methods in Developing Health Services and Computer Technology. Qual Health Res. januar 2007;17(1):122–30.

25. Engblad MA, Herstal EP, Wegener EK, Kayser L. Using an Empathetic Approach to Explore Technology Readiness and Needs for Digital Services to Assist People with Dementia. Int J Environ Res Public Health. 2. august 2024;21(8):1023.

26. Spradley JP. The ethnographic interview. New York: Holt, Rinehart and Winston; 1979. 247 s.

27. Graneheim UH, Lindgren BM, Lundman B. Methodological challenges in qualitative content analysis: A discussion paper. Nurse Educ Today. september 2017;56:29–34.

28. Braun V, Clarke V. One size fits all? What counts as quality practice in (reflexive) thematic analysis? Qual Res Psychol. 3. juli 2021;18(3):328–52.
