# Supplementary material for: Considerations When Designing Inclusive Digital Health Solutions for Older Adults Living With Frailty or Impairments
Source: JMIR Form Res. 2024 Oct 21;8:e63832. doi: 10.2196/63832 (PMC11535789; doi:10.2196/63832)
Supplement: Multimedia Appendix 5 [file formative_v8i1e63832_app5.docx]

In the implementation phase, we found it important to focus on how the developed services and technologies are harmonized and interoperable to ensure combability when expanding the socio-technical ecosystem. This is important to avoid creating or consolidating existing silo systems. In SMILE this was ensured through regular meetings with technical partners to ensure the use of common standards and interoperability. This process was informed by the European Health Data Space (EHDS) (1), and the international standard Fast health data interoperable resources (FHIR) for the development of a database structure to be used across the living labs (2,3). One of the aims of SMILE was to demonstrate transferability and cultural adaptation of the ECM model. The strength of using ECM for integration is that there is developed protocols for how to map activities, responsibilities, where individuals are located when executing their responsibilities (e.g. in-person or virtually), the available technologies and context (4,5). This is an extension of the principles from the user task context model (6). This was demonstrated in Norway, where it was an important factor that the Danish living lab with the PreCare project had developed a Blueprint that cover the aspects of implementing and running a sustainable healthcare model with digital services for older adults with LTHC’s (5).This served as a guide for the development of the Norwegian living-lab in 2021. Based on this experience we recommend that projects that aim to develop and implement service models, build on existing blueprints, or create a blueprint in collaboration with clinicians, vendors, administrators, patient representatives and health economists.

### Infrastructuring

To establish a new ecosystem, in e.g., in a living lab, new equipment needs to be acquired and the stakeholders involved need to reset their mindset. This change can be conducted as action research as seen in the PreCare project (5). This involves an agile approach with a ‘buy-in’ for the involved stakeholders. It can also be managed by an implementation group using the GO-TO model, be based on inspiration from the ‘normalization process theory’ (7) or the NASSS (non-adoption, abandonment, scale-up, spread, sustainability) (8). The focus should not only be on how the implementation can be done but also on whether it will influence processes outside the living lab e.g., the utilization of hospital or community services (6). If processes outside a living lab are not considered, scaling up the innovations developed in the living lab, can be difficult e.g., in the meeting with existing services and solutions. A way to mitigate this problem is to have both outside-in processes involving stakeholders and policy makers in the existing systems and inside-out activities with clear communication and dissemination strategies (9). In SMILE a part of the vertical infrastructuring process was ensured by participation of all partners in a work-package that addressed both dissemination and exploitation potentials. Here emphasis was on webinars and participation at international conferences targeting formal and informal caregivers and policy makers. The outside-in process will also contribute to the vertical integration, which should result in ownership by leaders at regional or national level, including healthcare organizations, insurance companies, and politicians. In Norway the vertical integration to ensure sustainability and learning from the living lab CEO’s and directors at all levels in the regional hospital and municipalities were involved. The regional Innovation center was also involved and had a representative in the projects advisory board. In Denmark, the vertical process involved participation of the CEO of PreCare, and the regional board of politicians who endorsed the project was involved in initial activities. The SMILE project and activities were also governed by the leaders of innovation and digitalization from PreCare. This resulted in scaling up some of the principles from the ECM model and PreCare in the region, however many innovative solutions are not yet fully implemented.

**References**

1. European Comission. European Health Data Space [Internet]. EU; Tilgængelig hos: https://health.ec.europa.eu/ehealth-digital-health-and-care/european-health-data-space_en

2. Ayaz M, Pasha MF, Alahmadi TJ, Abdullah NNB, Alkahtani HK. Transforming Healthcare Analytics with FHIR: A Framework for Standardizing and Analyzing Clinical Data. Healthcare. 13. juni 2023;11(12):1729.

3. Dullabh P, Hovey L, Heaney-Huls K, Rajendran N, Wright A, Sittig DF. Application Programming Interfaces in Health Care: Findings from a Current-State Sociotechnical Assessment. Appl Clin Inform. januar 2020;11(01):059–69.

4. Phanareth K, Vingtoft S, Christensen AS, Nielsen JS, Svenstrup J, Berntsen GKR, m.fl. The Epital Care Model: A New Person-Centered Model of Technology-Enabled Integrated Care for People With Long Term Conditions. JMIR Res Protoc. 16. januar 2017;6(1):e6.

5. Blueprint. PRECARE: The PreCare clinic - A sector-neutral, data-driven, and person-centred healthcare service [Internet]. 2024. Tilgængelig hos: https://smileehealth.eu/knowledge-and-learning-from-smile-precare/

6. Bødker S, Dindler C, Iversen OS. Tying Knots: Participatory Infrastructuring at Work. Comput Support Coop Work CSCW. april 2017;26(1–2):245–73.

7. May CR, Mair F, Finch T, MacFarlane A, Dowrick C, Treweek S, m.fl. Development of a theory of implementation and integration: Normalization Process Theory. Implement Sci. december 2009;4(1):29.

8. Abimbola S, Patel B, Peiris D, Patel A, Harris M, Usherwood T, m.fl. The NASSS framework for ex post theorisation of technology-supported change in healthcare: worked example of the TORPEDO programme [Internet]. BMC medicine; 2019. Tilgængelig hos: doi.org/10.1186/s12916-019-1463-x

9. Hesseldal L, Kayser L. Healthcare innovation – The Epital: A living lab in the intersection between the informal and formal structures. Qualitative Sociology Review. 2016;12(In print).
